# Gluten tolerance prevents oral sensitization with enzymatic or acid hydrolyzed gluten: A study in Brown Norway rats

**DOI:** 10.1371/journal.pone.0231139

**Published:** 2020-04-06

**Authors:** Charlotte Bernhard Madsen, Katrine Lindholm Bøgh

**Affiliations:** National Food Institute, Technical University of Denmark, Kgs. Lyngby, Denmark; Tulane University, UNITED STATES

## Abstract

**Background:**

There are several reports describing allergy to hydrolyzed wheat products. After a large outbreak in Japan it was established that sensitization was caused by skin contact with acid hydrolyzed gluten in soap. It is still not clear if other forms of hydrolyzed gluten may sensitize, and if the skin is the only relevant route of sensitization in humans and to what extent oral tolerance to wheat play a role.

**Objectives:**

The aim of the present study was to examine if wheat-tolerant rats may be sensitized via the oral or i.p. route when exposed to gluten, enzymatic or acid hydrolyzed gluten.

**Methods:**

Brown Norway rats, tolerant to wheat, were dosed by three i.p. injections without adjuvant or by oral gavage daily for 35 days with the three gluten products, respectively. Sera were analyzed by ELISA for specific IgG1 and IgE. In addition inhibition and avidity ELISAs were performed. Results were compared to a similar study in rats naïve to wheat.

**Results:**

More than half the animals had measurable IgG1 at the start of the dosing period. I.p. immunization resulted in significant specific IgG1 and IgE to the antigen used for immunization but significantly lower than in naïve rats. The results of inhibition and avidity ELISA’s indicate that the underlying tolerance to epitopes common to the three products influences the immune response. Oral dosing did not induce significant changes in response to either gluten or the hydrolyzed gluten product used for dosing.

**Conclusions:**

The study shows that i.p. immunization with the three products can break the underlying tolerance to wheat. Exposure by the oral route to enzymatic or acid hydrolyzed gluten is very unlikely to break an already established tolerance to gluten and induce sensitization.

## Introduction

Wheat is part of our normal diet. In addition, different forms of hydrolyzed wheat protein are used both in cosmetic and food products. Incidence of allergic reactions to food containing hydrolyzed wheat proteins (HWP) have prompted the question, how these subjects were sensitized, in particular the route of sensitization and the nature of the hydrolysates giving rise to allergic reactions [1, 2, 3, 4, 5].

Wheat contains hundreds of proteins. The majority of wheat proteins are gliadins and glutenins, collectively called gluten and per definition not soluble in water [6]. Gluten may be hydrolyzed by enzymes increasing solubility, or may be hydrolyzed by acid and heat, which in addition to increased solubility result in deamidation of the amino acids glutamine and asparagine. Depending on the degree of acid hydrolysis the functionality of the proteins changes [7, 8]. One of the characteristics of acid hydrolyzed gluten is its emulsifying properties, making it useful in food products, but also in cosmetics [9].

Pecquet [1] were the first to describe food allergy to HWP in an atopic woman that had used skin cream containing HWP. Based on the clinical history the authors suggested that the route of sensitization was through the skin. Other reports on food allergic reactions to HWP followed, but the method of hydrolysis and the route of sensitization was still not clear [2, 3]. In later cases of food allergy acid hydrolysed gluten (AHG), used as emulsifier, was identified as the elicitor, but again the route of sensitization was not identified [4,5].

Awareness of exposure to food via the skin as a possible route of sensitization to food allergens is relatively new. Sensitization to peanut via the skin, primarily in children with atopic dermatitis (AD), has been described to occur after exposure to peanut oil-based skin creams [10] or to household dust containing peanut protein [11].

The reports of sporadic cases of food allergic reactions to HWP including AHG were followed by a large outbreak in Japan, where AHG in a facial soap caused not only food allergy to AHG, but also to ordinary wheat products. Here it was clear that the route of sensitization to AHG as well as the breaking of oral tolerance to gluten was caused by skin exposure to the AHG [12,13,14]. In contrast, it is not known if sensitization in gluten tolerant subjects to HWP, may also be caused by oral exposure e.g. when AHG is used as an emulsifier in food.

To shed light on this, we have previously studied the sensitizing capacity of gluten (G), enzymatic (EHG) and acid hydrolyzed gluten (AHG) by oral exposure. In order to study the inherent sensitizing capacity this was done in Brown Norway (BN) rats naïve to gluten i.e. bred on a wheat-free diet for more than three generations. We found that G, EHG or AHG may sensitize rats that are naïve to gluten via both the oral and intraperitoneal (i.p.) route. The capacity to sensitize via the oral route was modest for all three products. The study also showed that EHG is immunological very similar to gluten and that acid hydrolysis gave rise to an immune response that suggests presence of new epitopes not present in native gluten [15].

In continuation hereof, the aim of the present study was to examine if rats having oral tolerance to wheat proteins including gluten, and hence resembling humans with oral tolerance to wheat in the diet, may be sensitized via the oral or i.p. route when exposed to G, EHG or AHG. This was done using the same protocol as used in the former study, allowing a comparison between responses in naïve and tolerant rats.

## Material and methods

### Ethics statement

Animal experiments were carried out at the National Food Institute’s facilities in Mørkhøj. Ethical approval was given by the Danish Animal Experiments Inspectorate. The authorization number given: 2009/561-1710. The experiments were overseen by the in-house Animal Welfare Committee.

### Wheat gluten products

Wheat gluten (G, unmodified), enzymatic hydrolyzed wheat gluten (EHG) and acid hydrolyzed wheat gluten (AHG) were provided by Tereos Syral, Aalst, Belgium. The characteristics of the products are as described in [15]. *In short*, *the enzyme hydrolysed gluten was hydrolysed with an endoprotease at neutral pH and moderate temperature*. *The acid hydrolysed gluten was hydrolysed without enzyme at pH below 2*.*5 and temperature above 80*^*°*^*C resulting in a degree of deamidation of 60%*.

### Preparation of soluble fractions of wheat gluten products for analysis of antibody responses

Gluten products were dissolved by carefully adding 1 mg/mL unmodified gluten or hydrolyzed gluten products to phosphate buffered saline (PBS; 137 mM NaCl, 3 mM KCl, 8 mM Na_2_HPO_4_, 1 mM KH_2_PO_4_; pH 7.2) before magnetic stirring for 2 hours at room temperature (RT) followed by ultrasonication for 3–5 hours. After centrifugation at 2000×*g* for 20 min. at 4°C, supernatants were collected and stored at -20°C.

### Animals

BN rats were from our in-house colony at the National Food Institute (DTU, Denmark). Rats were bred and kept for at least two generations on conventional rat chow containing wheat (Altromin 1314, with 6.73% protein originating from gluten, Altromin, Lage, Germany), in order to induce oral tolerance to wheat. During dosing they were kept on an in-house wheat-free diet [16]. Rats were housed in macrolon cages (two/cage) at 22 ± 1°C, relative humidity 55 ± 5%, air change 10 times/hour, and electric light from 9.00 am—9.00 pm. Diet and acidified water (pH 3.5) were given *ad libitum*. Animals were inspected twice daily and body weights recorded weekly. At termination of sensitization experiments all animals were anaesthetized by hypnorm-dormicum and sacrificed by exsanguinations.

### Animal sensitization studies

Sensitization was studied in two different rat models; an intraperitoneal (i.p.) model based on i.p. immunization with the soluble fraction of the gluten products and an oral model where rats were dosed by gavage with ‘whole’ gluten products suspended in water.

Positive control sera were produced as described in [15]. Sera from all animal experiments were stored at -20°C until analyses.

#### I.p. study with gluten products

After weaning, at the age of four weeks, rats were continued on the wheat containing diet for two weeks (to assure that gluten tolerance was established) and then moved to a wheat-free diet for two weeks before initiation of dosing (in theory, to have tolerant BN rats with as little gluten-specific IgG1 as possible to start with). A blood sample was drawn at day 0 before first dosing and again at day 35 at termination of the study. Groups of eight rats (four per sex) were dosed with G, EHG or AHG with i.p. injection day 0, 14 and 28. The dose used was 200 μg/animal/immunization.

#### Oral sensitization study with gluten products

Female BN rats bred on a wheat-containing diet for 2–3 generations were kept on a wheat containing diet 2–3 weeks after weaning to be sure to maintain the developed tolerance. At day 0 of the experiment rats were moved to a wheat-free diet and a blood sample was drawn. Female rats (12/group) were dosed by gavage for 35 days with 20 mg of G, EHG or AHG, dissolved in 0.5 mL PBS. Gluten was suspended in an aqueous acetic acid solution (pH = 4). Blood was drawn day 14 and 28 and at sacrificed day 42. Only female rats were used as more responders are found amongst female BN rats than among males when dosed by gavage [17].

### Enzyme-Linked ImmunoSorbent Assay (ELISA)

Serum samples from the sensitization studies were analyzed for specific IgG1 and IgE by ELISAs. Positive and negative serum control pools were included on each plate. Absorbance was measured at 450 nm with a reference wavelength of 630 nm, using a microtitre reader (Gen5, BioTek Instruments). Results are expressed as Log_2_ titer values and defined at as the interpolated dilution of the given serum sample leading to the mean absorbance for the negative control serum sample +3 SD.

#### Detection of specific IgG1 by indirect ELISAs

Detection of specific IgG1, was as described in [15] using plates coated with 100 μL/well of 2 μg/mL of the soluble fraction of unmodified gluten or hydrolyzed gluten products.

#### Inhibition ELISAs for examination of IgG1-binding capacity

Assay procedures and interpretations were as described in [15]. In short, serum samples from individual rats were diluted to reach a final OD between 0.8 and 1.0 and preincubated 1:1 for 1 hour at RT with serial 10-fold dilutions of the soluble fraction of gluten products (0.1 ng/mL to 100 μg/mL) before triplicates of serum/inhibitor mix (and serum samples with no inhibitor as a control) were added to the wells.

#### Detection of specific IgE by antibody-capture ELISAs

In order to be able to detect specific IgE in competition with the much higher level of IgG, an IgE capture ELISA was developed. Plates (96 well, Maxisorp, Nunc, Roskilde, Denmark) were coated overnight at 4°C with 0.5 μg/mL of mouse anti-rat IgE (HPMAB-123 HybriDomus, Cytotech, Hellebæk, Denmark) in carbonate buffer (15 mM Na2CO3, 35 mM NaHCO3; pH 9.6). Between each step, plates were washed five times in PBS containing 0.01% (v:v) Tween (PBS-T). After blocking of remaining active sites for 1 hour at 37°C with PBS-T containing 0.1% skimmed milk powder (SMP) (w:v) (G, AHG) or 1% SMP (w:v) for EHG, plates were incubated with serially two-fold diluted rat serum samples and then with Digoxigenin (DIG)-coupled gluten products. G-DIG was coupled 1:20 and diluted 1:2000; EHG-DIG was coupled 1:40 and diluted 1:1000 in PBS-T. AHG-DIG was coupled 1:20 and diluted 1:1000 in PBS-T with 0.1% SMP (w:v). Coupling of gluten products with DIG was essentially done as described in [18]. After washing, plates were incubated with sheep anti-DIG-POD (poly):HRP (ROCHE, Mannheim, Germany) diluted in PBS-T containing 0.1% SMP (w:v) (AHG only) (diluted 1:1000). Reaction was visualized by adding 100 μL/well of TMB-one substrate (4380A, Kementec Diagnostics, Taastrup, Denmark) for approximately 12 min and stopped with 100 μL/well of 0.2 M H_2_SO_4_.

In order to be able to compare the IgE results between gluten naïve and tolerant rats, sera from the study in naïve rats were re-analyzed using the same IgE capture ELISA as described above. The results were essentially the same as in the original study, but we have used the data from the re-analyzes in this paper.

#### Avidity measurements

For measurement of the binding strength between gluten products and IgG1 antibodies a thiocyanate inhibition ELISA based on the method described by El-Khouly [19] was conducted. Thiocyanate, a chaotropic agent, can disrupt the hydrogen bonding network between water molecules and hence interferes with non-covalent binding. As a result, antigen-antibody complexes are dissociated in a dose dependent manner. Plates were coated with 100 μL/well of 2 μg/mL antigen solution in carbonate buffer and incubated overnight at 4°C. Between each step, plates were washed five times in PBS-T. Serum samples were diluted in PBS-T to give an OD of approx. 1, and 50 μL/well were added in six rows of quadruplicates for each serum sample. After incubation for 1 hour at RT, 50 μL/well of potassium thiocyanate (KSCN, Sigma, St. Louis, MO, USA) diluted in PBS-T was added to the plates in increasing concentrations (0, 0.1, 0.2, 0.5, 1, 2, 4, 6, and 8 M) and incubated for 30 min at RT. For detection, 50 μL/well of HRP-labeled mouse-α-rat IgG1 (3060–05, Southern Biotech) diluted 1:20,000 in PBS-T was added to each well and incubated for 1 hour at RT. Reaction was visualized as described earlier.

Under the given assay conditions, it was determined that KSCN did not influence the binding of antigen to the plates.

Avidity results of individual serum samples are expressed as the concentration of KSCN required for inhibition of 50% (IC_50_) of antigen-antibody binding, defined as a 50% reduction of OD, so that the lower the concentration of KSCN needed for 50% inhibition the lower the avidity of antigen-antibody interactions. Calculations were performed as described by El-Khouly et al 2007.

Serum samples from animals dosed i.p. with gluten in the Kroghsbo study [15] were also tested.

### Statistical analyses

Curve analysis and statistical analyses were made using GraphPad Prism v8 (San Diego, CA, USA). Curves obtained from inhibitory ELISA were tested for variance with one-way ANOVA, Bartlett’s test for equal variances, and then Tukey’s multiple-comparisons test. No statistical significant variances between the curves were obtained, which enabled the calculation of IC50 values. The calculated IC50 and antibody titer values were examined for group differences using the non-parametric one-way ANOVA Kruskal-Wallis test followed by Dunn’s multiple-comparisons test to compare groups. Asterisks indicate statistically significant differences between the given groups: * p ≤ 0.05, ** p ≤ 0.01, *** p ≤ 0.001, and **** p ≤ 0.0001.

## Results

In the following the term naïve rats refers to results from the similar study in rats bred and raised on a wheat free diet for several generations and reported in [15].

### I.p. dosing induces a modest immune response in gluten tolerant rats

**Fig 1:**
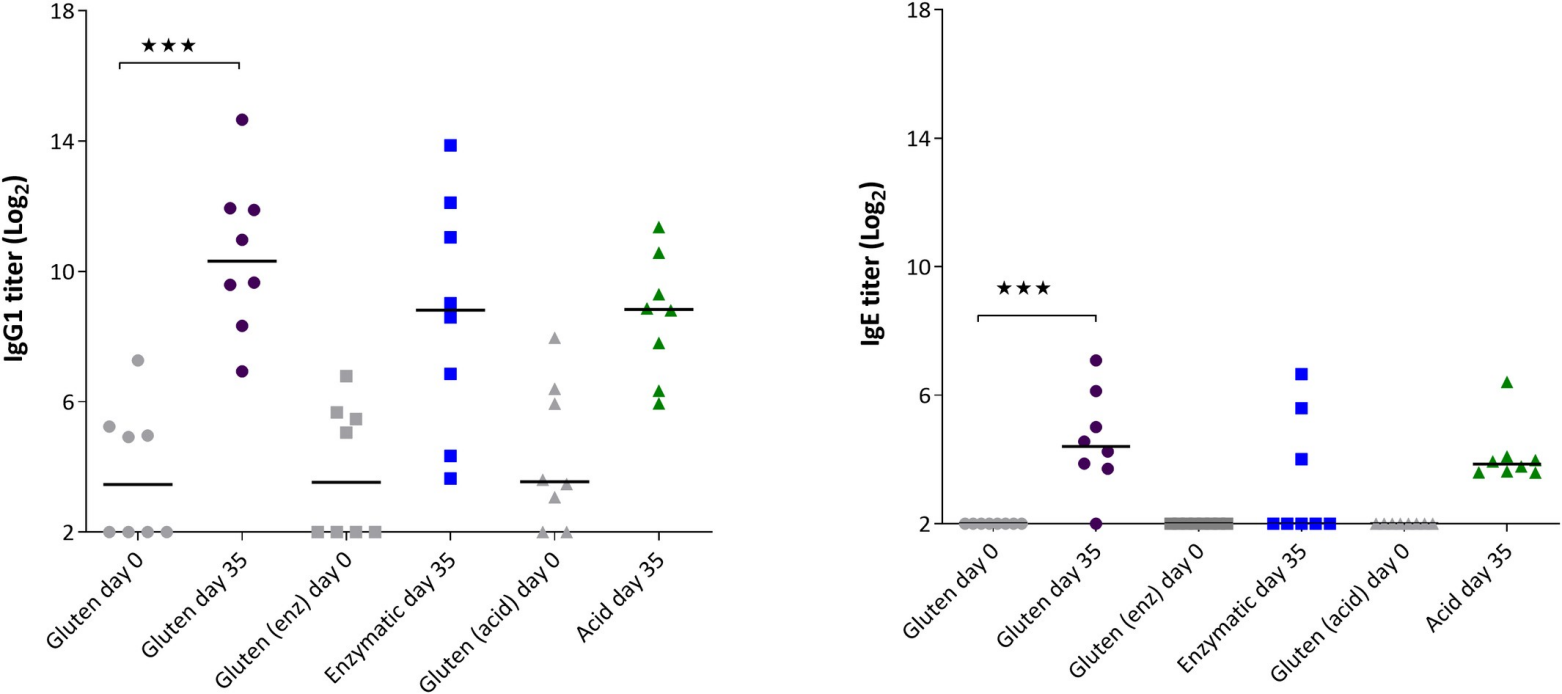
Specific antibody (IgG1 and IgE) responses to gluten at baseline (day 0) and to the respective antigens day 35 after three i.p. immunizations of gluten tolerant rats with gluten, enzyme or acid hydrolyzed gluten. *** p<0.001.

**Fig 2:**
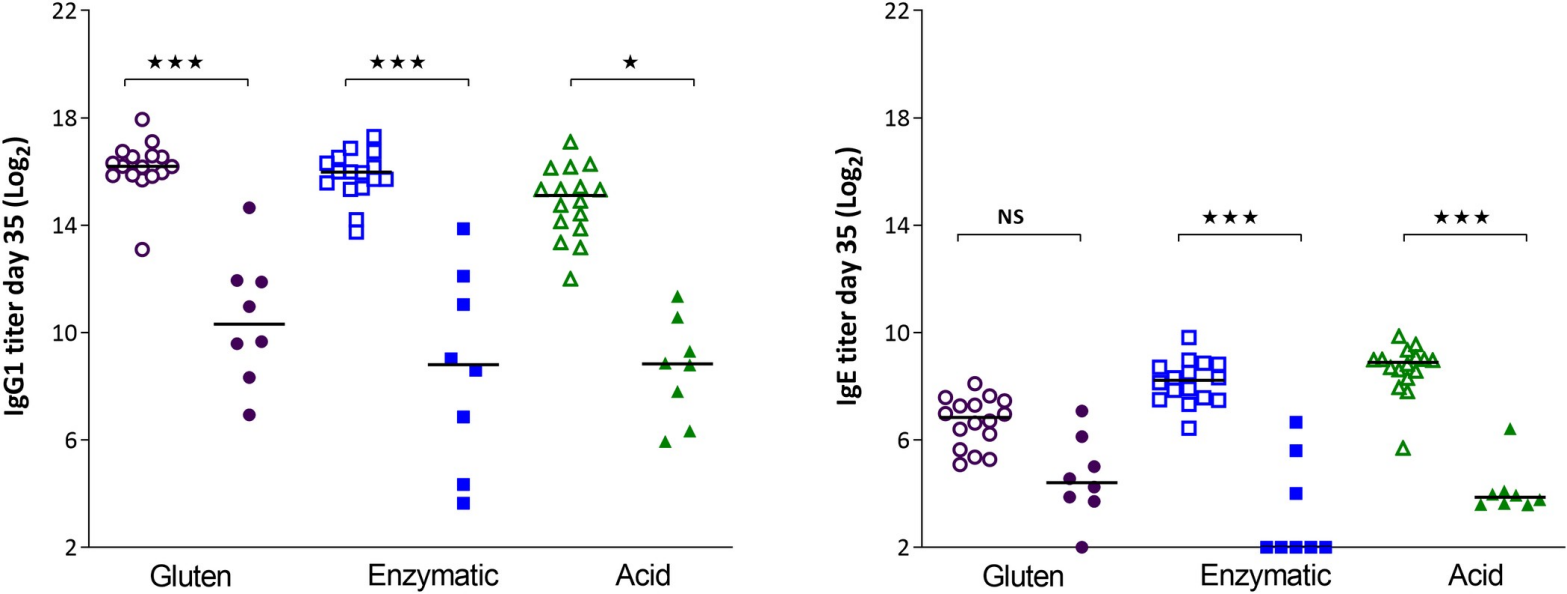
Specific antibody responses in gluten naïve rats (open symbols) and gluten tolerant rats (closed symbols) day 35 after three i.p. immunizations with gluten, enzyme or acid hydrolyzed gluten. * p<0.05, *** p<0.001.

As expected, some rats had a specific IgG1 response to G before initiating of the dosing regime, due to the gluten containing diet. I.p. immunization with G resulted in a statistically significant increase in the G-specific IgG1 and IgE in comparison to baseline. I.p. immunization with EHG or AHG also induced a significant IgG1 and IgE response to the respective antigens, which are though not statistically comparable to the baseline response to gluten as the assays measure antibodies to different antigens (Fig 1). For all three gluten products, the IgG1 responses were statistically significant lower than in naïve rats. For IgE there was only statistical significance for EHG and AHG (Fig 2). This shows, as expected, that tolerance to gluten decreases the immune response not only to gluten itself but also to the two hydrolyzed products both for IgG1 and IgE. In addition, it can be seen that there is a much larger variation in the response in tolerant rats compared to naïve rats (Fig 2).

To further characterize the response we performed inhibition ELISA and measured the binding strength (avidity) on individual serum samples.

### Inhibition ELISA indicates antibody responses to similar epitopes irrespective of the immunizing gluten product

**Fig 3:**
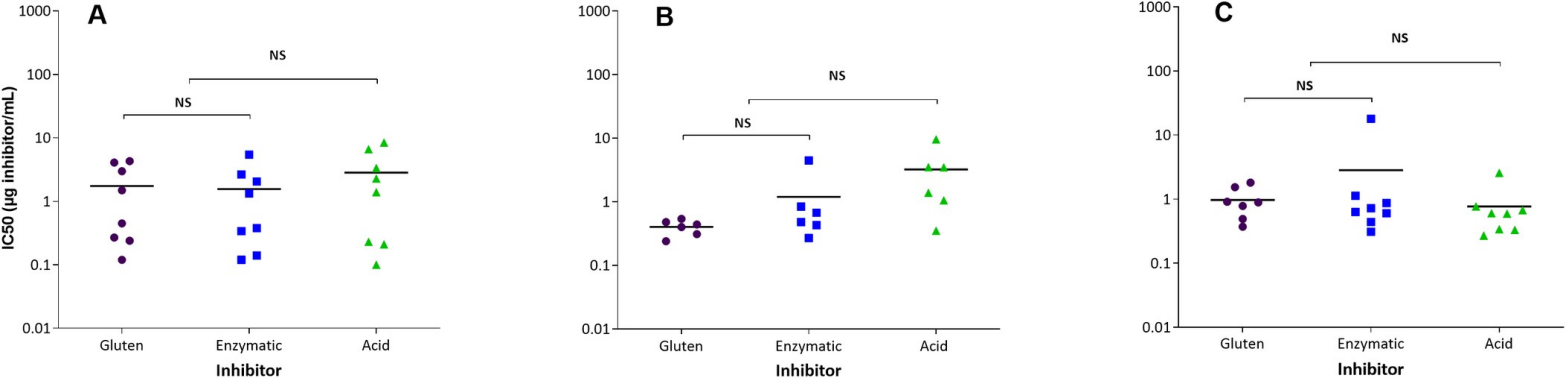
Inhibition ELISA. Sera from gluten tolerant rats immunized with Gluten (A), Enzymatic hydrolyzed gluten (B) or Acid hydrolyzed gluten (C) and inhibited with Gluten, Enzymatic or Acid hydrolyzed gluten.

**Fig 4:**
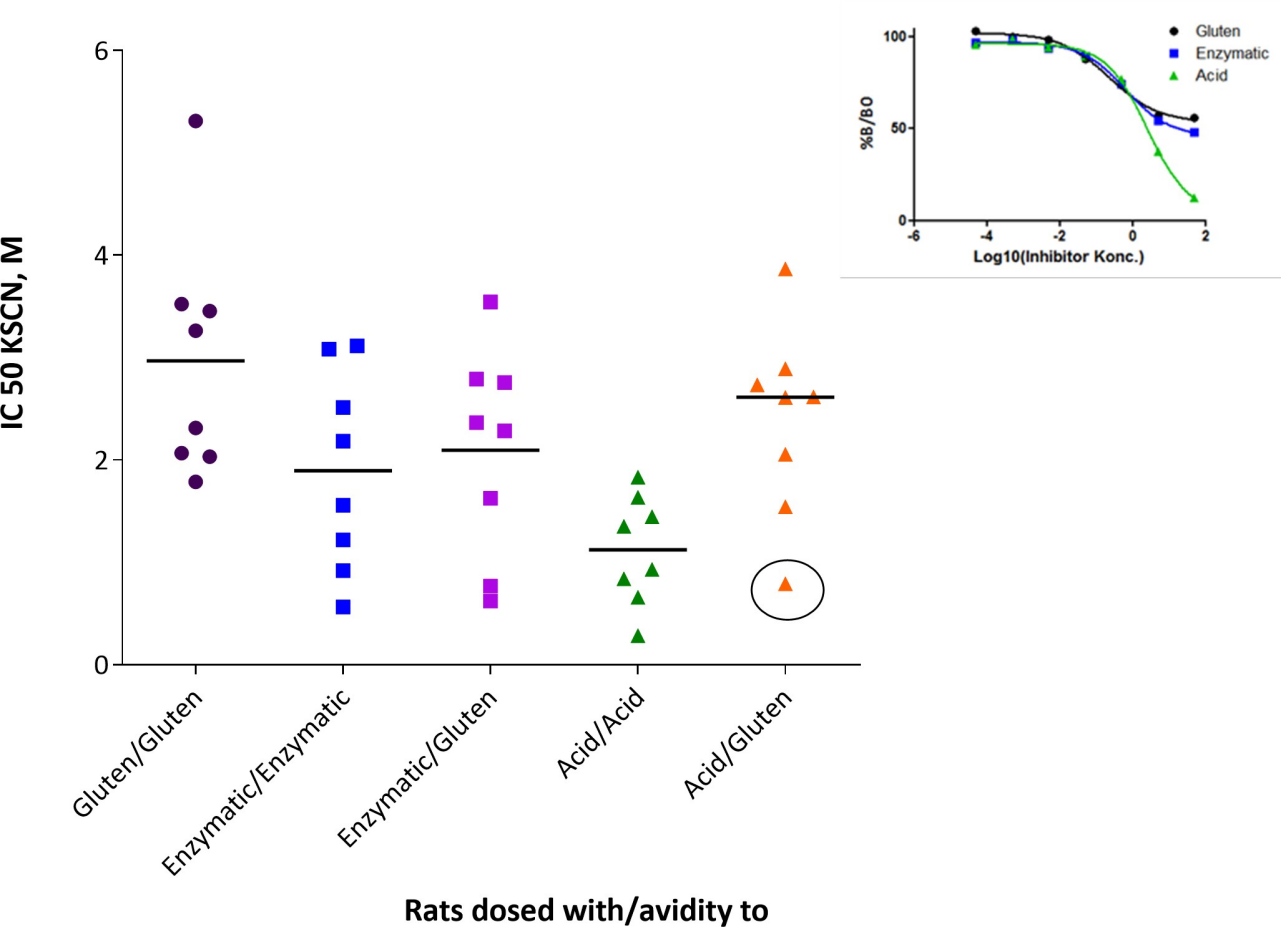
Avidity to the product used for immunization in gluten tolerant rats and to gluten in rats immunized with enzymatic and acid hydrolyzed gluten. The insert is the inhibition ELISA for the rat in the circle.

The inhibition ELISAs showed no significant differences between the products in their ability to inhibit the antibody responses, irrespective of whether antibodies were raised against G, EHG or AHG. Results varied between individual rats and there was no clear pattern. Fig 3 summarizes all results and shows IC50 i.e. the amount of antigen needed in order to inhibit 50% of the response. These results are in contrast to the results in naïve rats, where the variation in inhibition curves within the groups was small and it was evident that AHG was a poor inhibitor of G and EHG and vice versa [15]. There was only one of the AHG immunized gluten tolerant rats that showed a pattern identical to the naïve rats (insert in Fig 4). In other animals inhibition curves were similar for all three products, indicating a response dominated by antibodies against epitopes shared by all three products.

### Binding strength is highly variable and is influenced by tolerance

To further characterize the immune response we looked at the binding strength between antigen and antibodies (avidity).

**Fig 5:**
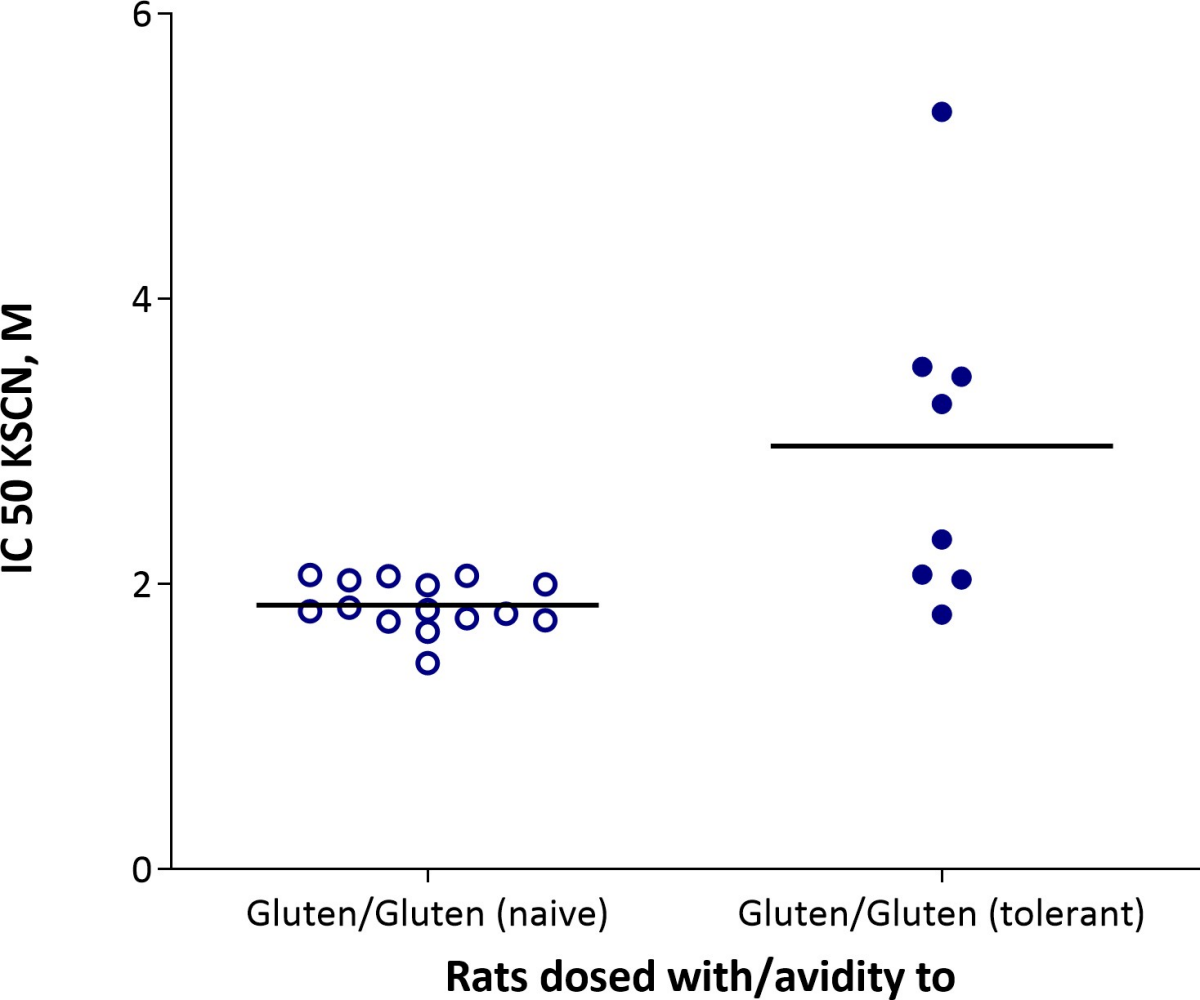
**Avidity to gluten in naïve (open symbols) or gluten tolerant rats (closed symbols) immunized i.p. with gluten.** The were no statistically significant differences.

The results showed a large variation in avidity within the groups (Fig 4). Sera from rats immunized with G had IgG1 with the strongest binding between antibodies and G. EHG induced antibodies with an intermediate binding between antibodies and both G and EHG. In contrast, AHG immunization resulted in an IgG1 response with an intermediate binding strength between antibodies and G but a low binding strength between antibodies and AHG. As avidity is the combined binding strength between antigens and antibodies these results cannot determine if the low binding to AHG is caused by low affinity antibodies or molecular changes in the epitopes of AHG. In addition, we compared avidity to gluten in naïve rats dosed with G from [15] with avidity from tolerant rats dosed with G (Fig 5). It can be seen that four of the tolerant rats develop antibodies with a higher avidity compared to the naive rats. These finding indicate that for some animals the underlying gluten tolerance have primed B cell maturation allowing further maturation upon i.p. immunization.

### Avidity is not correlated to antibody response at baseline

**Fig 6:**
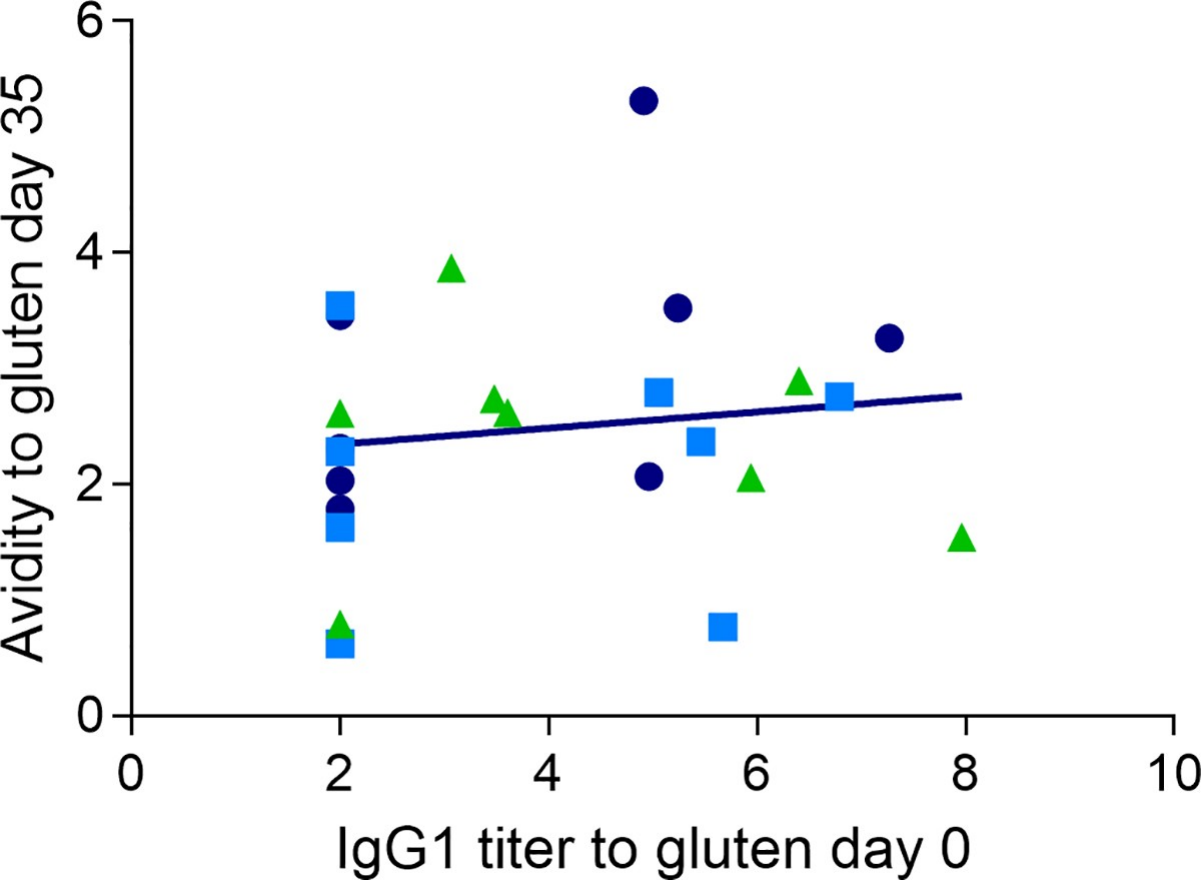
**Relation between specific IgG1 titer to gluten day 0 and avidity (IC50) to gluten day 35, after three i.p. doses of G (cirkels), EHG (squares) or AHG (triangles).** Each dot represents an animal. A titer of 2 is the cut-of value i.e. no measurable antibodies (p = 0.54).

As some of the animals had a measurable IgG1 titer to gluten at the start of the experiment (day 0), and others did not, we wanted to see if there was a correlation between the magnitude of the IgG1 titer to gluten at the start of the experiment, and avidity to gluten day 35. Fig 6 shows that there is no positive correlation between having a high IgG1 response to gluten day 0 and a high avidity to gluten after dosing with G, EHG or AHG. The same result is seen if the comparison is only made with animals dosed with gluten (data not shown). This indicates that tolerance was established day 0 whether it resulted in measurable IgG1 or not.

### Oral dosing does not sensitize

**Fig 7:**
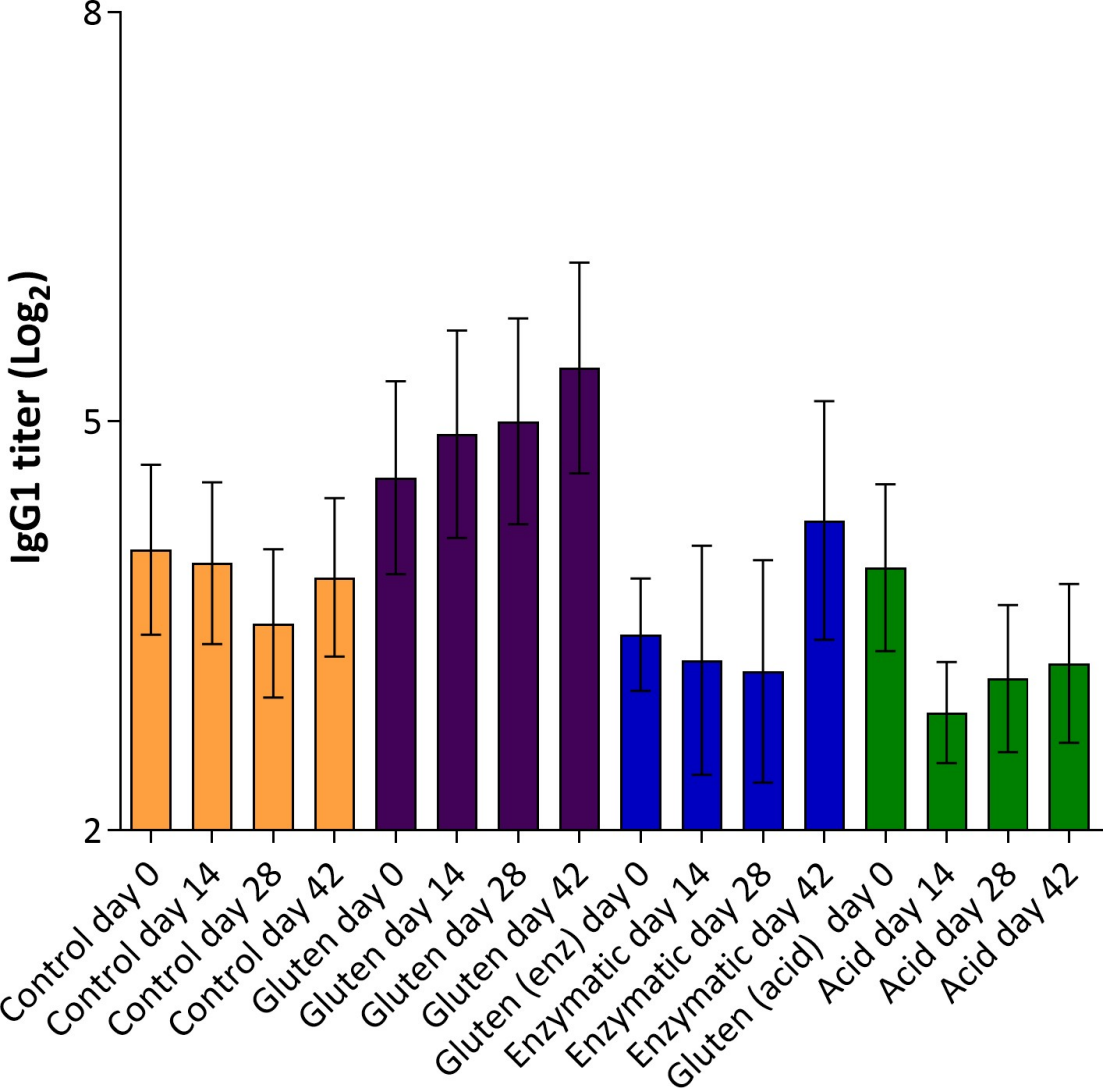
Specific antibody response to gluten at baseline (day 0) and to the respective antigens day 14, 28 and 42 after oral dosing for 35 days with no product (control), 20 mg gluten, enzymatic or acid hydrolyzed gluten. In the control group, antibodies to gluten were measured. Results are shown as mean with SEM. There were no statistically significant differences between groups.

Oral dosing with G, EHG or AHG had little effect on the levels of specific IgG1 that were remarkably constant from before dosing (day 0), during dosing at day 14 and 28 and one week after dosing day 42. There are no statistically significant differences between the groups (Fig 7). When comparing IgG1 levels at baseline with the IgG1 levels after oral gavage, for individual animals, some animals had a decrease in the specific IgG1 response and others had an increase. None of these changes were statistically significant (data not shown).

**Fig 8:**
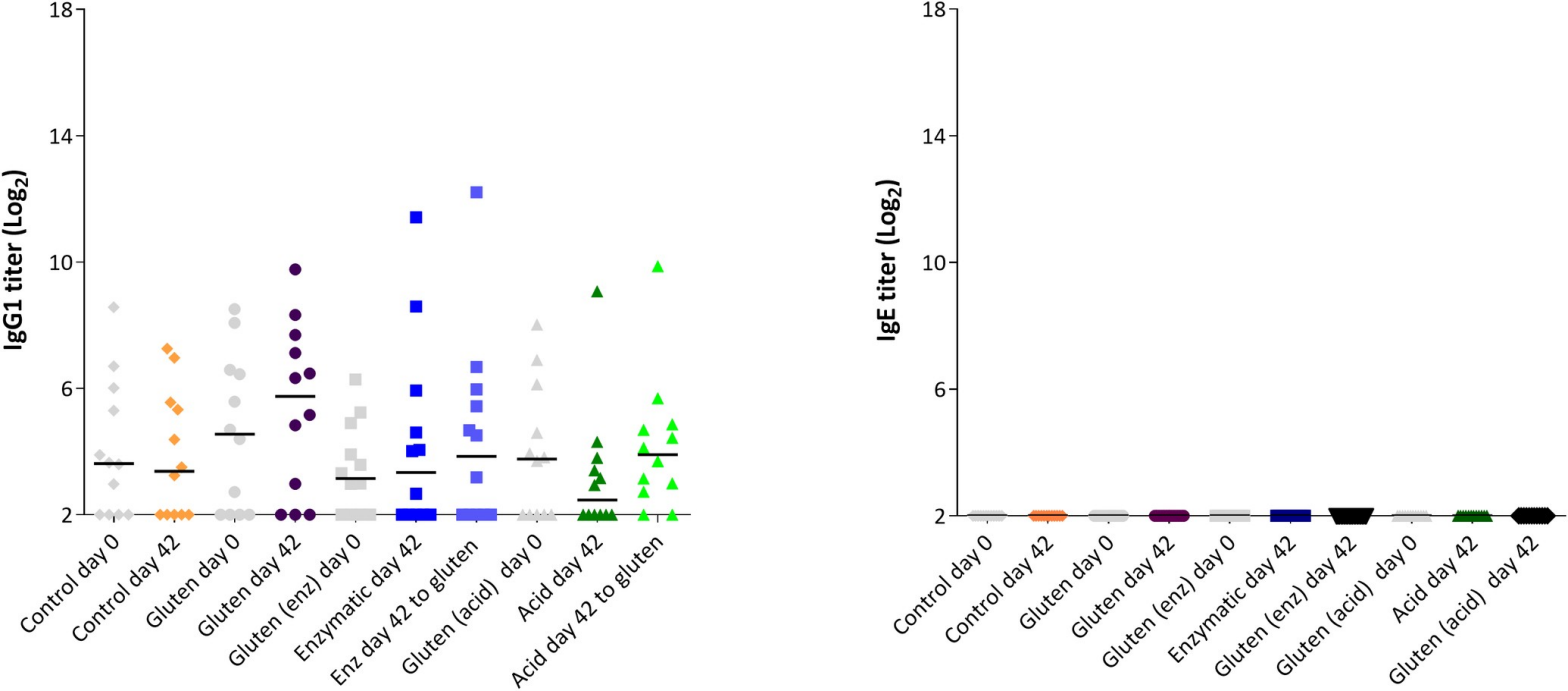
Specific antibody response to gluten at baseline, day 0 and 42, and to the respective antigens day 42 after oral dosing for 35 days with 20 mg gluten, enzymatic or acid hydrolyzed gluten. Results are shown as individual animals. There were no statistical differences between groups.

Fig 8 shows the specific IgG1 and IgE responses day 0 and day 42 to the antigen used for dosing (day 42) and in addition to gluten (day 0 and 42). The level of antibodies specific to the product used for dosing and to gluten is similar, with no statistical significant differences.

**Fig 9:**
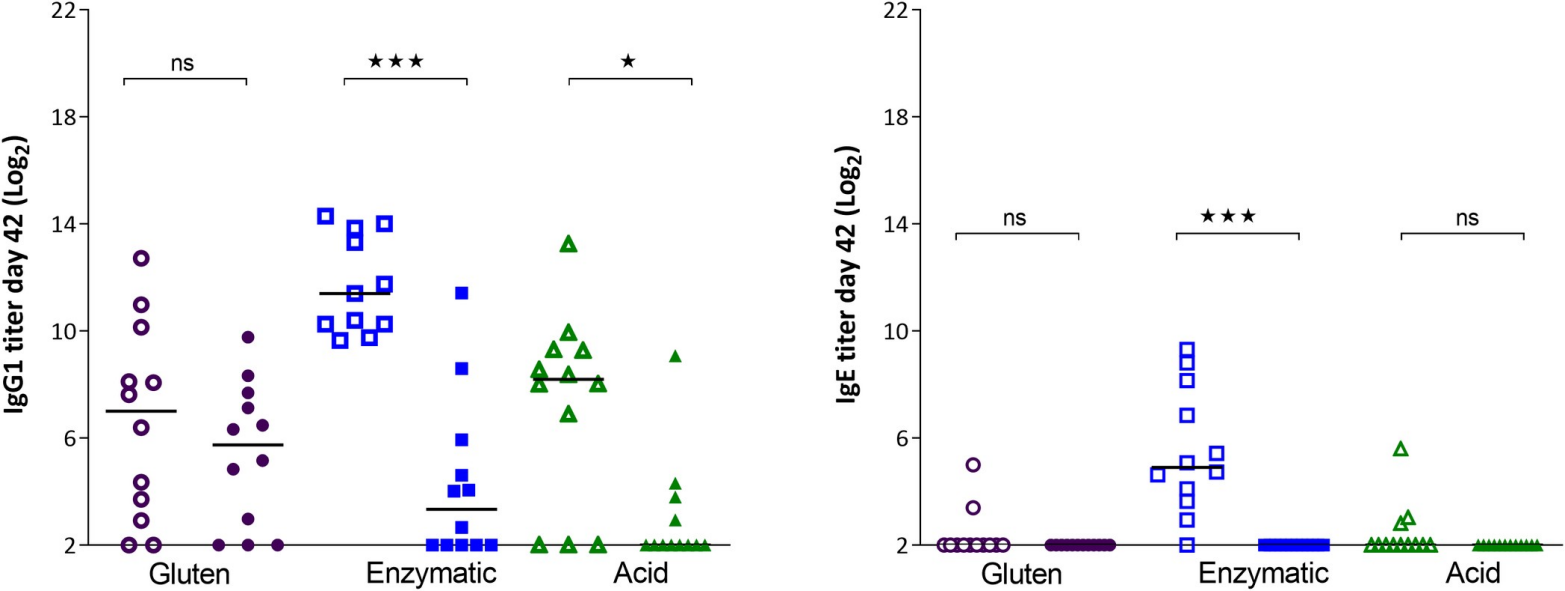
Specific antibody response in gluten naïve rats (open symbols) and gluten tolerant rats (closed symbols) after oral dosing for 35 days with 20 mg gluten, enzymatic or acid hydrolyzed gluten. * p<0.05, *** p<0.001.

When comparing the specific antibody responses after oral dosing in gluten naïve or gluten tolerant rats, it can be seen that the antibody response is lower in the tolerant rats. This difference is statistically significant for EHG and AHG (IgG1) and EHG (IgE) (Fig 9).

We have not performed inhibition ELISA and avidity studies in sera from the oral study, due to the low titer values.

### Specific IgG1 in serum is long lived

In the i.p. study rats were kept on the wheat-containing diet for two weeks after weaning and then moved to wheat-free diet for two weeks before dosing began, to try to minimize specific IgG1 to gluten at baseline. In the oral dosing study, the rats were kept on a wheat-containing diet for 2–3 weeks after weaning and the dosing began immediately after rats were moved to a wheat-free diet, to make sure the tolerance did not disappear. The results show that specific IgG1 response to gluten is remarkably constant in the non-dosed control rats and even in the oral dosed animals. There were no statistically significant differences in the specific IgG1 to gluten at baseline (day 0) in the two experiments despite the difference in weeks on wheat-free diet before day 0 blood samples (data not shown).

## Discussion

In the present study, we have shown that rats bred on a wheat-containing diet develop oral tolerance to gluten that may or may not result in measurable specific IgG1. Oral tolerance is the default response to food antigens in the gastrointestinal tract and is a state of systemic unresponsiveness. When these rats were dosed i.p. with G, EHG or AHG the tolerance could be broken resulting in induction of specific IgE antibodies. However, the level of specific IgE depended on the specific gluten product, where G and AHG resulted in a larger number of IgE responders than EHG. Further, i.p. immunization with the gluten products resulted in induction of specific IgG1 antibodies, shown by a significant increase in specific IgG1 antibodies compared to baseline.

When the response to i.p. dosing in tolerant rats were compared to the naïve rats it was seen that tolerance reduced the magnitude of the response statistically significantly for IgG1 and IgE (EHG and AHG), but increased the heterogeneity.

The increase in heterogeneity was also seen in the inhibition ELISA, where there was no consistent pattern within the groups, again in contrast to the study in naïve rats [15].

The underlying tolerance to gluten may have resulted in memory B cells capable of producing antibodies to various epitopes. When these B cells are activated by i.p. immunzation, antibodies with high affinity can be formed, because memory B cells are a result of affinity maturation [20]. The influence of tolerance varies between different animals. In the G dosed animals, half of the animals (4/8) developed antibodies of high avidity. The other half, developed antibodies with avidity similar to those produced in naïve rats i.e. as if there had been no affinity maturation or as if antibodies were formed to new epitopes. This response is not dependent of the specific IgG1 level day 0.

In the EHG and AHG immunized gluten tolerant animals the avidity of the antigen-antibody binding was also very heterogeneous, probably influenced by the underlying tolerance to gluten, where some animals in general were shown to have antibodies of high affinity and others of low. Further, the binding strength seemed to differ depending on the specific gluten product. Sera from rats sensitized with AHG showed lower avidity to AHG than to G, although AHG was an efficient inhibitor in the AHG inhibition ELISA. These results could be explained by the fact that AHG contains new epitope as a result of the acid hydrolysis process, which are different from those present on the EHG and G, due to the deamidation process. As the antibodies developed against the new epitopes have not gone through the same affinity-maturation process as the one that are shared between AHG and G and EHG, these epitope contributes to the lower avidity seen for antibodies raised against AHG and binding to AHG compared to binding to G. That the same pattern is not seen for the inhibitory ELISA is probably a matter of the inhibitory ELISA in addition to avidity also is dependent on the antibody specificity and clonality.

The inhibition ELISA showed that antibodies to the new epitopes were less prominent in tolerant rats compared to naïve rats. This was seen in the IgG1 response in the AHG immunized rats when sera from these rats were inhibited with G or EHG. G and EHG could effectively inhibit serum from 7/8 tolerant rats immunized with AHG. In the naïve rats this was only the case in 1/16 AHG immunized rats. These results can probably also be explained by the affinity-maturation process, in naïve rats, where epitopes on G, EHG and AHG are all new to the immune system, with no underlying tolerance and thereby no memory B cells. In contrast, in the tolerant rats, epitopes shared with G have undergone affinity-maturation.

In the present study, no statistically significant differences could be found between specific IgG1 or IgE responses to G, EHG and AHG. This is in contrast to a study by [21] where mice were i.p. immunization with native or deamidated gliadin (corresponding to AHG) and alum as adjuvant. They found that deamidated gliadin induced a higher IgE response and higher histamine release after challenge than native gliadin. The symptom scores after challenge were not different, though. The study also showed that the response to native gliadin had more type 1 features than the response to deamidated gliadin. The mice used in that study were bred on a wheat-containing diet and only kept on a wheat-free diet from three weeks before initiation of the experiment, and thus resembling our tolerant rats. The authors, however, do not discuss their results in light of the underlying tolerance. The degree of deamidation is similar in the two studies, but the massive use of a type 2 adjuvant is in contrast to the present study. Using the i.p. route surpasses the gastro-intestinal tract and thus resembles skin exposure more than it resembles oral exposure. Adachi [22] sensitized mice via the skin with G and AHG using sodium dodecyl sulphate (SDS) to enhance skin penetration. AHG was a better sensitizer than G and sensitization was increased by concurrent SDS exposure simulating soap. The mice were kept on ordinary mice feed that may have contained wheat. The possible influence of tolerance to wheat was not discussed in that paper. A study by Ballegaard [23] sheds light on the influence of tolerance on sensitization via the skin. BN rats that were either naïve or tolerant to gluten were sensitized on slightly damaged skin with G or AHG. After skin exposure animals were post-immunized orally, twice. The results showed that AHG is a more potent sensitizer than G in both naïve and tolerant rats, but that the IgE response was significantly higher in naïve animals.

The studies in animals indicate, that the new epitopes in AHG developed after deamidation, where glutamine residues are converted to glutamic acid residues, may well be the most important feature that renders AHG capable of sensitizing wheat tolerant individuals to AHG, but also break an already established tolerance to wheat via skin exposure to AHG as observed in Japanese patients [12, 13, 14]. *These results are supported by a study of IgE binding from patients with allergy to AHG, where IgE from serum reacted strongest with an epitope where two, three or four glutamines were substituted with glutamic acid mimicking different degrees of deamidation [24].*

In contrast, dosing G, EHG or AHG by oral gavage for 35 days does not induce a significant increase in immune response compared to baseline level, neither for IgG1 nor for IgE.

When the IgG1 response was followed over time, it was remarkably constant also in the non-dosed control animals, telling us that specific IgG1 once formed stays in serum for a long time.

When the results from the oral studies in naïve and tolerant rats are compared, it can be seen that the IgG1 response to EHG and AHG and the IgE response to EHG was significantly lower in the tolerant rats. The lack of difference in IgE to G and AHG is due to the very low response in both studies.

In conclusion, the present study showed that i.p. immunization of gluten tolerant rats with G, EHG or AHG induced a specific IgG1 and IgE response of similar magnitude. We have shown that gluten tolerance influence the binding strength of the response induced by subsequent immunization. The common epitopes on G and AHG makes it possible to have an AHG induced immune response to G. If the specific IgG1 response can be used as surrogate for the specific IgE response this could be a parallel to what happened in Japan where skin exposure to AHG could break gluten tolerance and result in allergic reactions to gluten [25].

In contrast, oral dosing with G, EHG or AHG in gluten tolerant rats had no effect on the level of specific IgG1 and IgE to none of the product used for dosing, or on the level of specific IgG1 and IgE to G. The study shows that exposure by the oral route to EHG or AHG is very unlikely to break an already established tolerance to gluten and induce sensitization.
